# Focal Adhesion Kinase Alleviates Simulated Microgravity-Induced Inhibition of Osteoblast Differentiation by Activating Transcriptional Wnt/β-Catenin-BMP2-COL1 and Metabolic SIRT1-PGC-1α-CPT1A Pathways

**DOI:** 10.3390/ijms26041669

**Published:** 2025-02-15

**Authors:** Yiling Bai, Zhaojia Wu, Scot C. Leary, Chen Fang, Michelle Yu, Harald Genth, Yufeng Xie, Jinhui Shi, Jim Xiang

**Affiliations:** 1 Cancer Research, Saskatchewan Cancer Agency, Saskatoon, SK S7N 4H4, Canada; lightbyl@163.com (Y.B.); zhaojia.wu@usask.ca (Z.W.); fangchensuda@163.com (C.F.); michelle.yu360@gmail.com (M.Y.); 2 Department of Oncology, University of Saskatchewan, Saskatoon, SK S7N 5E5, Canada; 3 Department of Biochemistry, Microbiology and Immunology, University of Saskatchewan, Saskatoon, SK S7N 5E5, Canada; scot.leary@usask.ca; 4 Institute of Toxicology, Hannover Medical School, D-30625 Hannover, Germany; genth.harald@mh-hannover.de; 5 Department of Thoracic Surgery, The First Affiliated Hospital of Soochow University, Suzhou 215006, China; sdxyf@163.com; 6 Department of Orthopedics, The First Affiliated Hospital of Soochow University, Suzhou 215006, China

**Keywords:** µg, osteoblast differentiation, FAK, Wnt/β-catenin, SIRT1, PGC-1α, CPT1A, mitochondrial biogenesis, FAO, CNF1

## Abstract

The metabolic poise, or balance, between glycolysis and fatty acid oxidation (FAO) has recently been found to play a critical role in osteogenic differentiation and homeostasis. While simulated microgravity (SMG) is known to impede osteoblast differentiation (OBD) by inhibiting the Wnt/β-catenin pathway, how it affects osteoblast metabolism in this context remains unclear. We previously analyzed the effect of SMG on the differentiation of pre-osteoblast MC3T3-E1 cells and found that it reduced focal adhesion kinase (FAK) activity. This, in turn, downregulated Wnt/β-catenin and two of its downstream targets critical for OBD bone morphogenic protein-2 (BMP2) and type-1 collagen (COL1) formation, leading to a reduction in alkaline phosphatase (ALP) activity and cell matrix mineralization. In this study, we further analyzed how SMG-induced alterations in energy metabolism contribute to the inhibition of OBD in MC3T3-E1 cells. Consistent with our earlier findings, we demonstrated that SMG inhibits OBD by downregulating the collective activity of FAK and the Wnt/β-catenin-BMP2-COL1 transcriptional pathway. Interestingly, we observed that SMG also reduces the abundance of sirtuin-1 (SIRT1), peroxisome proliferator-activated receptor-γ coactivator-1α (PGC-1α) and carnitine palmitoyl transferase-1α (CPT1A), which are all key metabolic factors regulating mitochondrial number and FAO capacity. Accordingly, we found that the mitochondrial content and FAO potential of MC3T3-E1 cells were lower upon exposure to SMG but were both rescued upon administration of the FAK activator cytotoxic necrotizing factor-1 (CNF1), thereby allowing cells to overcome SMG-induced inhibition of OBD. Taken together, our study indicates that the metabolic regulator SIRT1 may be a new target for reversing SMG-induced bone loss.

## 1. Introduction

During spaceflight, astronauts are exposed to many stressors that seriously affect their health [1]. Among these stress factors, aerospace microgravity (AMG) poses various physiological challenges to different biological systems, one of the most significant of which is inhibition of osteoblast differentiation (OBD). Impaired OBD in turn reduces bone mass and induces osteopenia [2,3] in a manner analogous to patients with osteoporosis [4,5].

Bone is maintained through a remodeling process that reflects the balance between bone resorption by osteoclasts and bone formation by mesenchymal stem cells (MSCs), osteoblasts and osteocytes [6]. The Wnt/β-catenin pathway plays an important role in OBD and, by extension, in the formation and regeneration of bone [4]. Wnt/β-catenin signaling downregulates the activity of glycogen synthase kinase-3β, leading to hypophosphorylation of β-catenin [4]. This posttranslational modification stabilizes β-catenin, which accumulates in the cytosol and then translocates into the nucleus [7]. Nuclear-localized β-catenin interacts with the transcription factors T-cell factor-1 (TCF1) and lymphoid enhancer-binding factor (LEF), the concerted activity of which upregulates the expression of the osteoblast maturation markers bone morphogenic protein-2 (BMP2) and type-1 collagen (COL1). These Wnt/β-catenin-dependent effects on osteoblast maturation characteristics are typically measured by alkaline phosphatase (ALP) activity and mineralization analyses [4,8,9].

Bone metabolism has recently been recognized as an underappreciated player in bone remodeling and homeostasis [10], with the metabolic poise of bone cell populations greatly affecting their potential for osteogenic differentiation [6]. For example, mitochondrial oxidative phosphorylation (OXPHOS) plays a critical role in the osteogenic differentiation of MSCs [11,12], and fatty acid oxidation (FAO) promotes OBD by stimulating β-catenin acetylation [11,13]. The NAD+-dependent histone deacetylase sirtuin-1 (SIRT1) deacetylates peroxisome proliferator-activated receptor-γ (PPARγ) coactivator-1α (PGC-1α) [14], promoting its activity as a transcription factor coactivator. PGC-1α activation, in turn, stimulates mitochondrial biogenesis by enhancing the expression of numerous nuclear-encoded mitochondrial gene products [15,16] such as carnitine palmitoyl transferase-1α (CPT1A), a key regulator of FAO, and glycerol channel aquaporin-9 (AQP9), an organelle transporter that imports glycerol for fatty acid esterification and triacylglycerol synthesis [17,18,19]. Thus, SIRT1 and PGC-1α are both critical regulators of mitochondrial content and FAO potential [14,15,16], and SIRT1 has been found to promote OBD and bone deposition [20,21,22].

Simulated microgravity (SMG) is a ground-based method mimicking AMG that was developed to study the effects and molecular mechanisms by which AMG alters OBD. SMG inhibits osteogenesis in both pre-osteoblast MC3T3-E1 [23,24] and MSC CH310T1/2 cell lines [25,26]. The osteogenic differentiation of MSCs is suppressed by altering energy metabolism [27], and SMG has been shown to inhibit OBD by impairing FAO [28]. Similarly, Liu et al. reported that SMG impaired osteogenic differentiation in CH310T1/2 MSCs by inhibiting SIRT1 and OXPHOS [29], while Dai et al. demonstrated that this phenotype could be rescued upon activation of SIRT1 and restoration of OXPHOS capacity [30]. However, how SMG affects osteoblast energy metabolism and how this may contribute to impaired OBD is less well studied.

We previously investigated the effect of SMG on pre-osteoblast MC3T3-E1 cell differentiation [31]. We demonstrated that SMG reduced focal adhesions (FAs) and focal adhesion kinase (FAK) activity and downregulated Wnt/β-catenin signaling. In turn, this lowered the activity of its downstream targets, TCF1, BMP2, COL1 and ALP, all of which are crucial for OBD, and led to a reduction in cell matrix mineralization in vitro and tibial trabecular bone loss in vivo [31]. Here, we analyzed how SMG impinges upon energy metabolism and contributes to the inhibition of OBD in MC3T3-E1 cells. We demonstrate that in addition to inhibiting FA formation and downregulating FAK signaling, SMG reduces mitochondrial content and FAO capacity. We further show that these metabolic effects are driven by the downregulation of SIRT1, PGC-1α, CPT1A and AQP9 expression, which, along with the repression of the transcriptional Wnt/β-catenin-BMP2-COL1 pathway, could be rescued by administering the FAK/RhoA activator cytotoxic necrotizing factor-1 (CNF1) [31,32,33].

## 2. Results

### 2.1. SMG Inhibits Focal Adhesions

**Figure 1:**
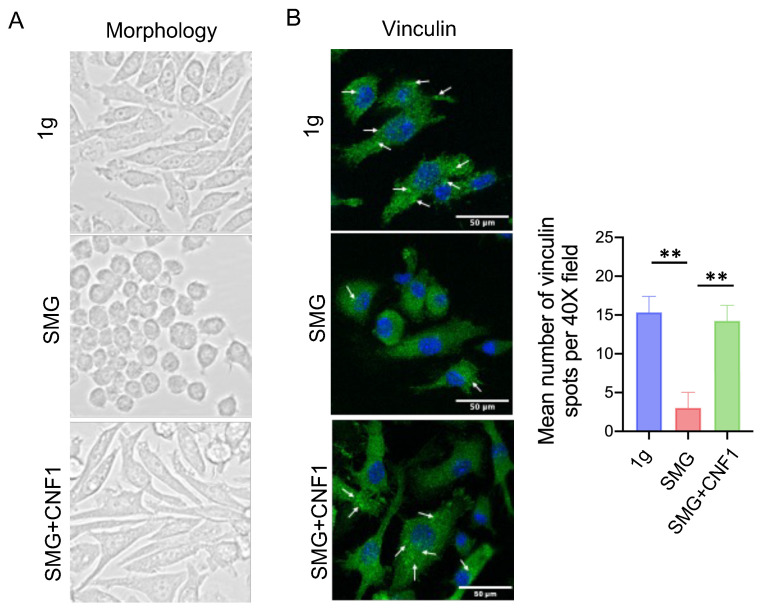
SMG alters the cytoskeleton and inhibits FAs. Cells cultured under 1 g, SMG or SMG + CNF1 conditions were imaged (**A**) by light microscopy to show morphology and (**B**) by fluorescence microscopy to show FITC-vinculin-positive (green) FA spots (white arrows). Cells were also counterstained with DAPI (blue) to visualize nuclei. The histogram bars represent the average number of FA spots detected using ImageJ software (v1.45) from three random regions under a 40× objective lens magnification field. Scale bar: 50 μm. Data from three independent experiments are presented as the mean ± SD. Significance analysis of the experimental data for each group was performed using Student’s *t* test (** *p* < 0.01).

To assess the effect of SMG on FAs, we cultured pre-osteoblast MC3T3-E1 cells on chamber slides under ground (1 g) and SMG conditions. After 2 d, MC3T3-E1 cells cultured under SMG showed a rounded-like morphology (Figure 1) due to alterations in their cytoskeletal structures, as we have previously described [31]. Because the integrin-binding protein vinculin is involved in recruiting FAK to FAs, we stained MC3T3-E1 cells cultured under 1 g and SMG with an anti-vinculin antibody and DAPI to label nuclei. We then used fluorescence microscopy to visualize and quantify vinculin-positive FAs (white arrows) [31] and observed that their abundance was significantly reduced in cells cultured under SMG compared to those cultured under 1 g (Figure 1). Thus, SMG dramatically impairs the formation of cellular FAs.

### 2.2. SMG Suppresses the Activity of FAK and the Transcriptional Wnt/β-Catenin-BMP2-COL1 Pathway Critical for OBD

**Figure 2:**
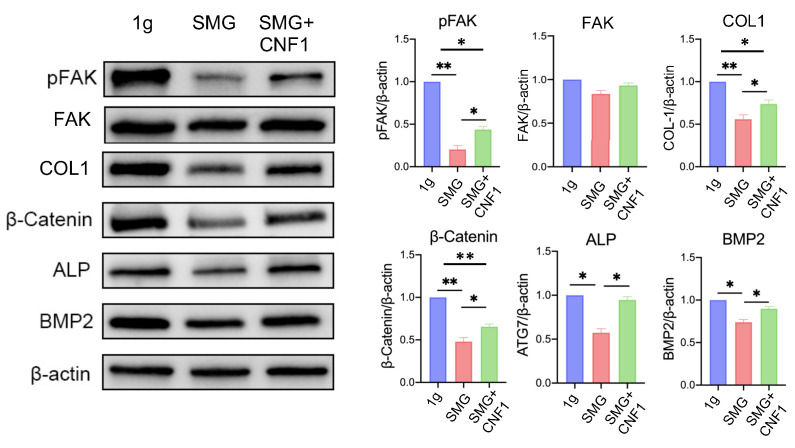
SMG attenuates OBD by inhibiting the Wnt/β-catenin signaling pathway. Western blot analysis of the steady-state levels of pFAK (Y397), COL1, β-catenin, ALP and BMP2 in MC3T3-E1 cells cultured under 1 g, SMG or SMG + CNF1 conditions. The histogram bars represent protein abundance normalized to the β-actin control and expressed relative to MC3T3-E1 cells cultured under the 1 g condition. Data from three independent experiments are presented as the mean ± SD. Significance analysis of the experimental data for each group was performed using Student’s *t* test (* *p* < 0.05; ** *p* < 0.01).

To further investigate the effect of SMG on the activity of FAK and the transcriptional Wnt/β-catenin signaling pathway, we performed Western blot analyses using whole-cell lysates derived from MC3T3-E1 cells cultured under 1 g and SMG conditions. Consistent with our previous findings [31], SMG significantly reduced the abundance of the active, phosphorated form of FAK (Y397) and downregulated Wnt/β-catenin signaling as well as the steady-state levels of its downstream targets BMP2 and COL1 (Figure 2). These data collectively emphasize that SMG inhibits the activity of several regulatory inputs critical for OBD.

### 2.3. SMG Inhibits Osteoblast ALP Activity and Mineralization

**Figure 3:**
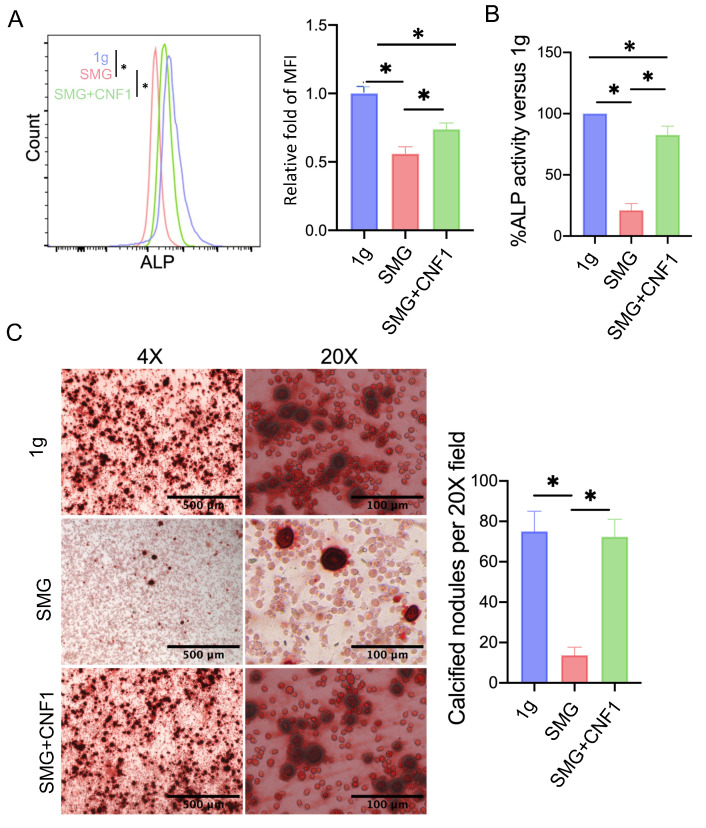
SMG inhibits OBD and mineralization. (**A**) MC3T3-E1 cells cultured under 1 g, SMG or SMG + CNF1 conditions were stained with ALP and analyzed by flow cytometry to quantify its abundance. The histogram bars represent the mean fluorescence intensity (MFI) for each group relative to MC3T3-E1 cells cultured under the 1 g condition. (**B**) Analysis of ALP activity in MC3T3-E1 cells cultured under SMG and SMG + CNF1 conditions compared to 1 g. (**C**) Representative images of MC3T3-E1 cells stained with Alizarin Red after 14 d of culture to depict OBD. The calcified nodules per 20× field were quantified using ImageJ software. Scale bar: 500 μm or 100 μm. For all panels, data from three independent experiments are presented as the mean ± SD. Significance analysis of the experimental data for each group was performed using Student’s *t* test (* *p* < 0.05).

Osteoblasts produce ALP, an enzyme critical for the calcification and mineralization of bones [34]. Therefore, we used lysates derived from MC3T3-E1 cells cultured under 1 g and SMG conditions to measure ALP abundance and activity as well as mineralization as direct readouts of osteoblast maturation [23]. We found that the relative abundance of Wnt/β-catenin-regulated ALP was reduced roughly two-fold in MC3T3-E1 cells cultured under SMG (Figure 3A). Consistent with this finding, SMG inhibited OBD and bone deposition by significantly reducing ALP activity (Figure 3B) and mineralization (Figure 3C).

### 2.4. SMG Reduces the Abundance of the Key Metabolic Regulators SIRT1, PGC-1α, CPT1A and AQP9

**Figure 4:**
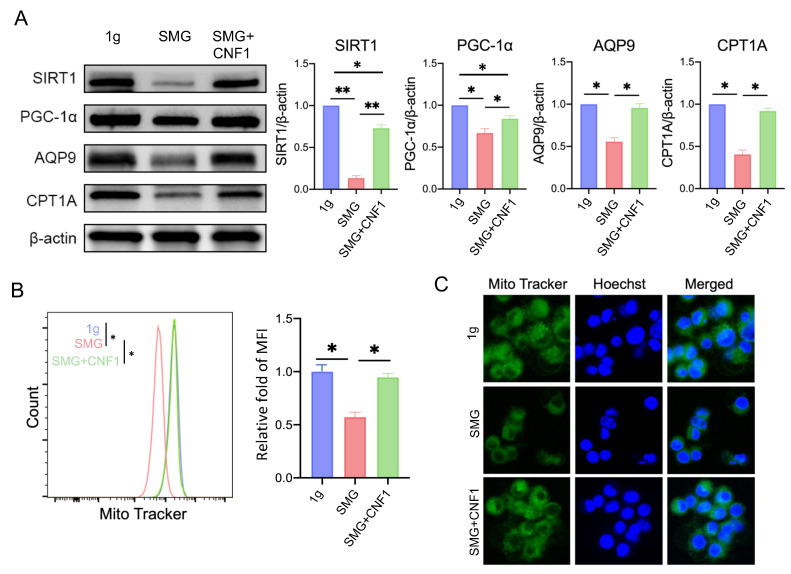
SMG impairs mitochondrial biogenesis by inhibiting the SIRT1-PGC-1α-CPT1A signaling pathway. (**A**) Western blot analysis of SIRT1, PGC-1α, AQP9 and CPT1A abundance in MC3T3-E1 cells cultured under 1 g, SMG or SMG + CNF1 conditions. The histogram bars represent protein abundance normalized to the β-actin control and expressed relative to MC3T3-E1 cells cultured under the 1 g condition. (**B**) MC3T3-E1 cells cultured under 1 g, SMG or SMG + CNF1 conditions were stained with the mitochondria-specific dye MitoTracker Green FM and analyzed by flow cytometry to quantify organelle mass. The histogram bars represent the mean fluorescence intensity (MFI) for each group relative to MC3T3-E1 cells cultured under the 1 g condition. (**C**) Representative confocal images of MitoTracker green-labeled mitochondria to show organelle content in MC3T3-E1 cells cultured under 1 g, SMG or SMG + CNF1 conditions. Cells were also stained with Hoechst solution to label nuclei in blue. For panels (**A**,**B**), data from three independent experiments are presented as the mean ± SD. Significance analysis of the experimental data for each group was performed using Student’s *t* test (* *p* < 0.05; ** *p* < 0.01).

To investigate whether SMG altered the expression of the metabolic regulators SIRT1, PGC-1α, CPT1A and AQP9, we performed Western blot analyses using whole-cell lysates derived from MC3T3-E1 cells cultured under 1 g and SMG conditions. When compared to MC3T3-E1 cells cultured under 1 g condition, SMG significantly reduced the steady-state levels of all four metabolic regulators and, in particular, those of the histone deacetylase SIRT1 (Figure 4A).

### 2.5. SMG Lowers the Mitochondrial Content of MC3T3-E1 Cells

Mitochondria are multifunctional organelles that produce energy to support cellular homeostasis and, by extension, functional activity and survival [11,12,13]. To investigate the impact of SMG on mitochondrial number, we stained MC3T3-E1 cells with the mitochondrial dye MitoTracker Green and analyzed them by flow cytometry (Figure 4B) and confocal microscopy (Figure 4C). Both analyses revealed that cells cultured under SMG had lower mitochondrial content than those cultured under the 1 g condition.

### 2.6. SMG Reduces the Reliance of MC3T3-E1 Cells on FAO

**Figure 5:**
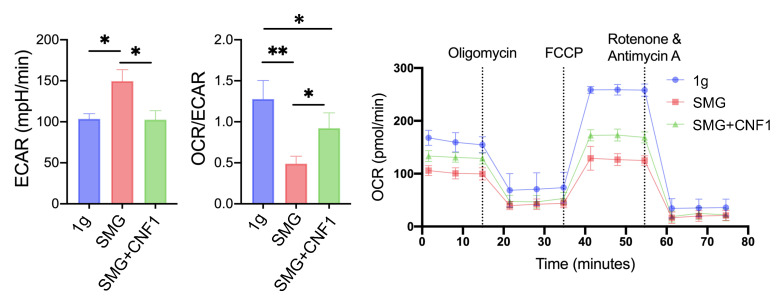
SMG reduces FAO metabolism. Bar graphs of the ECAR and OCR/ECAR ratio as well as the OCR measured in real time and in response to the indicated mitochondrial inhibitors in MC3T3-E1 cells cultured under 1 g, SMG or SMG + CNF1 conditions. Vertical dotted lines indicate the time when a total of 1.5 µM oligomycin, 2.5 µM FCCP, and 0.5 µM rotenone and antimycin-A were sequentially added to the wells. Data from three independent experiments are presented as the mean ± SD. Significance analysis of the experimental data for each group was performed using Student’s *t* test (* *p* < 0.05; ** *p* < 0.01).

To assess energy metabolism in MC3T3-E1 cells cultured under SMG and 1 g conditions, we used the Seahorse assay platform to analyze their bioenergetic profiles. Measurements were made as previously described [17,18,19] under basal conditions or upon the addition of inhibitors that specifically block ATP synthesis (oligomycin), uncouple electron transport chain (ETC) activity from ATP synthesis (FCCP) or ablate electron flux through ETC complexes I and III (rotenone, antimycin A). MC3T3-E1 cells exposed to SMG had an elevated extracellular acidification rate (ECAR), indicative of an increased reliance on glycolytic metabolism, and a significantly lower oxygen consumption rate (OCR) and reduced FAO compared to cells cultured under 1 g (Figure 5). These results are consistent with a previous report [35] and indicate that MC3T3-E1 cells dramatically shift their fuel preference from FAO to glycolysis for ATP generation in response to SMG.

### 2.7. CNF1 Treatment Activates FAK and Alleviates SMG-Induced Inhibition of OBD by Stimulating the Transcriptional Wnt/β-Catenin-BMP2-COL1 and Metabolic SIRT1-PGC-1α-CPT1A Pathways

The E. coli toxin CNF1 is an activator of RhoA and FAK signaling factors [32,33,36,37]. To assess whether FAK activation counteracts the ability of SMG to inhibit the Wnt/β-catenin and SIRT1 pathways, we included an additional treatment group in all of our experiments in which MC3T3-E1 cells exposed to SMG were also treated with 30 ng/mL of CNF1, as we have previously described [31,32,33]. Interestingly, CNF1 treatment rescued the FA defect induced by SMG (Figure 1). CNF1 treatment also partially restored the abundance of phospho-FAK (pFAK) and β-catenin, and that of their downstream osteoblast maturation markers COL1 and BMP2 (Figure 2). Consistent with these observations, CNF1 treatment overcame the ability of SMG to repress ALP expression (Figure 3A) and activity (Figure 3B) and promoted mineralization in MC3T3-E1 cells (Figure 3C). Finally, CNF1 treatment partially or completely restored the abundance of key metabolic factors (Figure 4A) and normalized mitochondrial content (Figure 4B,C), reverting the SMG-induced fuel preference from glycolysis to FAO (Figure 5). Taken together, our data indicate that CNF1-induced FAK activation alleviates SMG-induced inhibition of OBD by restoring the activity of the transcriptional Wnt/β-catenin-BMP2-COL1 and metabolic SIRT1-PGC-1α-CPT1A pathways.

## 3. Discussion

In modern cellular and molecular biology, two basic characteristics have been well defined: (i) the differentiated cellular phenotype and biological function and (ii) the metabolic fuel preference required to provide energy in support of homeostasis. Importantly, the coupling of both characteristics is indispensable for proper cellular function. For example, we recently demonstrated that the pro-survival cytokines IL-7 and IL-15 promote CD8+ memory T (TM)-cell formation through the coupled activation of the transcriptional FOXO1-TCF1-Id3-Eomes and metabolic AMPK-ULK1-ATG7-PGC-1α pathways [17,18]. Depletion of either the transcriptional regulator FOXO1 or the metabolic regulator AMPK in IL-7- or IL-15-treated CD8+ T cells attenuates T-cell function [17,18,38], indicating that the coordinated activity of both pathways is indispensable for T-cell memory formation.

**Figure 6:**
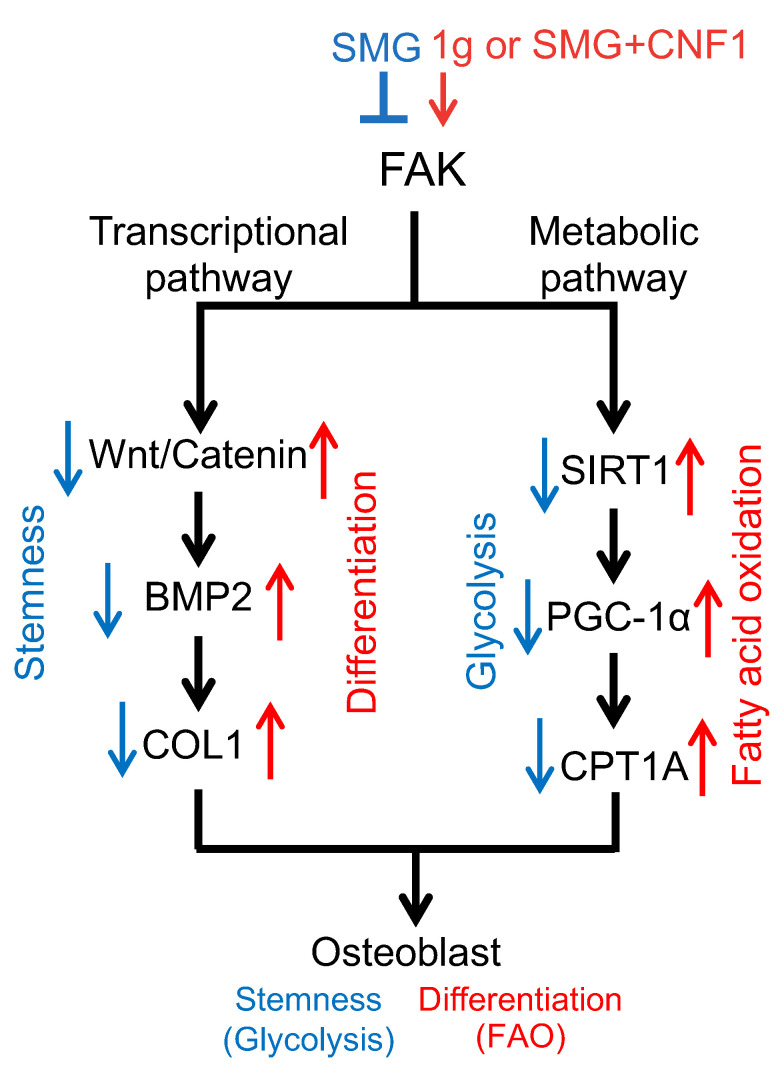
SMG modulates OBD by regulating the activity of both transcriptional and metabolic pathways. Schematic diagram showing that SMG inhibits FAK and downregulates FAK-regulated signaling pathways, thereby leading to the inhibition of OBD. These FAK-regulated signaling pathways include the transcriptional Wnt/β-catenin-BMP2-COL1 pathway for OBD and the metabolic SIRT1-PGC-1α-CPT1A pathway responsible for generating adequate ATP from FAO. The FAK activator CNF1 reverts the suppressive effects of SMG via the activation of the transcriptional Wnt/β-catenin-BMP2-COL1 pathway and the metabolic SIRT1-PGC-1α-CPT1A pathway. In this figure, red arrows represent upregulation and blue arrows denote downregulation.

In this study, we investigated how SMG affects OBD by modulating both transcriptional and metabolic pathways in the mouse pre-osteoblast cell line MC3T3-E1. We demonstrated that SMG (1) inhibits FA formation and reduces ALP activity and mineralization; (2) downregulates FAK signaling and the transcriptional Wnt/β-catenin-BMP2-COL1 pathway; and (3) reduces mitochondrial content and FAO by repressing the metabolic SIRT1-PGC-1α-CPT1A pathway. These cumulative effects allow SMG to maintain pre-osteoblast cells in a static state of stemness whereupon they rely on glycolysis for energy production (Figure 6). Because SIRT1, OXPHOS and FAO promote OBD and bone formation [6,11,12,13,20,21,22], we assume that the transcriptional Wnt/β-catenin-BMP2-COL1 and metabolic SIRT1-PGC-1α-CPT1A pathways are both indispensable with respect to the regulation of OBD in MC3T3-E1 osteoblast cells.

Cell surface integrins interact with the extracellular matrix at contact sites called FAs, which are bound by the cytoskeleton and heterodimers of α- and β-integrins to form cellular systems that sense and respond to external physical and chemical signals [39]. These FAs are composed of various macromolecules that collectively form FA complexes containing (i) α- and β-integrin transmembrane receptors, (ii) intracellular adaptor proteins (IAPs), such as the scaffolds talin, vinculin and paxillin, and (iii) IAP-recruited signaling molecules such as FAK and ras homolog gene (Rho) family GTPases [40]. Among these constituents, FAK plays a central role, serving as a scaffold for other FA components and a critical signaling initiator for various pathways via its binding to Src and paxillin [41].

The *E. coli* toxin CNF1 binds to the cell laminin receptor, leading to its internalization and transfer to the cytosol, where it catalyzes the reversible activation of the Rho family GTPases [36] that control gene transcription, actin cytoskeleton organization and cell proliferation, migration and survival [42]. We previously demonstrated that CNF1 also activates FAK, and that FAK activation reverses the SMG-induced inhibition of tumor cell proliferation and metastasis by modulating the mTORC1 and AMPK pathways [32]; overcomes SMG-promoted cell apoptosis via the mTORC1/NF-κB and ERK1/2 pathways [33]; and results in SMG-inhibited OBD via the transcriptional Wnt/β-catenin-BMP2-COL1 pathway [31]. In this study, we found that the ability of CNF1 to reverse the effects of SMG on OBD in MC3T3-E1 cells also requires the metabolic SIRT1-PGC-1α-CPT1A pathway (Figure 6).

Taken together, we demonstrated that FAK activation alleviates SMG-induced inhibition of OBD by coordinately upregulating the activity of the transcriptional Wnt/β-catenin-BMP2-COL1 and metabolic SIRT1-PGC-1α-CPT1A pathways. Therefore, our study indicates that in addition to conventional targeting of transcriptional Wnt/β-catenin signaling [43], the metabolic factor SIRT1 may represent a new target for the development of novel therapeutics aimed at preventing SMG-induced bone loss or treating patients with osteoporosis.

## 4. Materials and Methods

### 4.1. Cells, Antibodies and Reagents

The MC3T3-E1 mouse pre-osteoblast cell line was obtained from Thermo Fisher Scientific (Rockford, IL, USA) and grown in α-modified minimum essential medium (α-MEM; HyClone, Logan, UT, USA) supplemented with 10% fetal bovine serum (FBS; Gibco, Gaithersburg, MD, USA) and gentamicin (Gibco, Gaithersburg, MD, USA). Primary antibodies against PGC-1α (sc13067) and β-actin (sc8432) were purchased from Santa Cruz Biotechnology (Dallas, TX, USA). Primary antibodies against SIRT1 (9475), β-catenin (8480), pFAK (pFAK-Y397) (3283), vinculin (13901) and COL1 (72026) were obtained from Cell Signaling Technology (Danvers, MA, USA). Primary antibodies against AQP9 (ab191056), CPT1A (ab128568) and BMP2 (ab214821) were obtained from Abcam (Cambridge, MA, USA). A primary antibody against ALP (AF2910) was purchased from R&D Systems (Minneapolis, MN, USA). Horseradish peroxidase (HRP) and fluorescein isothiocyanate (FITC)-conjugated goat anti-rabbit secondary antibodies (31460, 32460) were purchased from Thermo Fisher Scientific (Rockford, IL, USA). The ProlongTM Gold Antifade Mountant with the DNA stain DAPI was obtained from Life Technologies Inc. (Carlsbad, CA, USA). Ascorbic acid, β-glycerol phosphatase, dexamethasone and Alizarin Red were obtained from Sigma-Aldrich (Darmstadt, Germany). *E. coli* toxin CNF1, which activates both FAK and RhoA, was obtained from Dr. Harald Genth (Hannover Medical School, Germany) [32,33].

### 4.2. Clinostat for SMG and Cell Culture

The SMG environment was simulated by a three-dimensional clinostat random positional machine (RPM) from the Center for Space Science and Applied Research (Chinese Academy of Sciences, Beijing, China), as previously described [31]. To examine the gravitational effect, MC3T3-E1 cells were plated into T25 culture flasks or chamber culture slides (Nalgene Nunc International Inc., Rochester, NY, USA) and grown at 37 °C in a 5% CO_2_ incubator for 24 h to allow the cells to attach. Cells were then cultured for 2 d on the clinostat under SMG conditions in 37 °C culture medium. Control cells were placed in the incubator under ground conditions (1 g). MC3T3-E1 cells cultured under SMG were also treated with CNF1 (30 ng/mL), as previously described [31]. The selected CFN1 dose (30 ng/mL) was based on previous culture studies [44,45] as well as unpublished observations by Dr. Genth. To assess the effect of SMG on OBD, MC3T3-E1 cells were grown for 14 d on chamber culture slides filled with osteoblast maturation medium [α-MEM medium supplemented with ascorbic acid (5 µg/mL), β-glycerophosphate (10 mM) and dexamethasone (0.1 µM)], sealed and placed on the clinostat under SMG conditions at 37 °C in a CO_2_ incubator. The medium was changed every 3 d, with ALP activity measured after 7 d and matrix mineralization analyzed after 14 d, as previously described [42].

### 4.3. Fluorescence Microscopy

For FA immunofluorescence staining, MC3T3-E1 cells cultured on chamber slides under 1 g, SMG or SMG + CNF1 conditions were fixed with 4% paraformaldehyde for 15 min, permeabilized with 0.1% Triton X-100 for 15 min and blocked with 5% FBS in phosphate-buffered saline (PBS) for 30 min at room temperature. The permeabilized cells were then incubated overnight at 4 °C with rabbit anti-vinculin antibody (1:100) in PBS containing 5% FBS and 0.05% Triton-X-100. The cells were washed with PBS and incubated for 1 h at room temperature in PBS containing secondary FITC-labeled anti-rabbit antibody. After rinsing three times with PBS, the plastic chambers were removed, and the cells were stained using ProlongTM Gold Antifade Mountant with DNA stain DAPI and covered by glass cover slides, as previously described [31]. Cell morphology was imaged under bright light and FITC-vinculin was visualized by fluorescence microscopy (EVOS 5000, Thermo Fisher Scientific, Rockford, IL, USA). A minimum of three random regions under the 40× objective lens magnification field were imaged for each cell group. Green spots of vinculin were counted in each region.

### 4.4. Western Blot Analysis

MC3T3-E1 cells cultured under 1 g, SMG or SMG + CNF1 culture conditions were lysed in ice-cold RIPA buffer supplemented with a protease and phosphatase inhibitor cocktail (Thermo Fisher Scientific, Rockford, IL, USA) and left on ice for 30 min. The cell lysates were centrifuged at 4 °C for 30 min at 12,000 rpm, and the protein concentration of the clarified extract was determined using a PierceTM BCA Protein Assay Kit (Thermo Fisher Scientific, Rockford, IL, USA). Each protein sample (30 µg) was separated on a 4–20% sodium dodecyl sulfate–polyacrylamide gel electrophoresis (SDS-PAGE) gradient gel and transferred to a polyvinylidene difluoride (PVDF) filter membrane (Bio-Rad, Hercules, CA, USA). The membranes were blocked at room temperature for 1 h using 5% bovine serum albumin (BSA) in PBS and 0.1% Tween 20 (PBST). The membranes were then washed three times for 5 min per wash with PBST and incubated overnight at 4 °C with primary antibodies against proteins of interest. Membranes were subsequently exposed to another PBST wash step, incubated at room temperature for 1 h with HRP-labeled secondary antibodies and then washed with PBST again. Immunoreactive bands were visualized using a ChemiDoc MP imaging system (Bio-Rad, Hercules, CA, USA) as previously described [31]. Their intensity was then quantified using ImageJ Software (TreeStar, Ashland, OR, USA) and normalized to β-actin intensity to account for any differences in loading.

### 4.5. Flow Cytometry

ALP staining was performed by incubating cells for 30 min on ice in PBS containing ALP primary antibody, followed by another 30 min incubation at room temperature in the dark with FITC-conjugated goat anti-rabbit secondary antibody in PBS. The cells were then washed three times and resuspended in PBS supplemented with 2% fetal calf serum (FCS) and 0.1% sodium azide for flow cytometry analyses. For mitochondrial analyses, organelle mass was analyzed by flow cytometry after staining cells with 100 nM MitoTracker Green for 15 min at 37 °C and washing them three times with PBS supplemented with 2% FCS and 0.1% sodium azide. Flow cytometry data were acquired using a CytoFLEX (Beckman Coulter, Brea, CA, USA) and analyzed with FlowJo software (v10.10, TreeStar, Ashland, OR, USA).

### 4.6. ALP Activity Analysis

MC3T3-E1 cells were cultured for 7 d in osteogenic medium under 1 g, SMG or SMG + CNF1 culture conditions to assay ALP activity. Cells were washed twice with PBS and lysed for 30 min on ice in RIPA buffer. ALP activity was measured with an ALP Assay Kit (Abcam, Cambridge, MA, USA) and a microplate reader (Thermo Fisher Scientific, Rockford, IL, USA) that measured absorbance change. Relative ALP activity in the SMG or SMG + CNF1 samples was normalized to total cellular protein, which was measured with a BCA Protein Assay Kit, prior to being expressed as a percentage of ALP activity in the control 1 g sample, as previously described [31].

### 4.7. Alizarin Red Staining

MC3T3-E1 cells were cultured for 14 d in osteogenic medium under 1 g, SMG or SMG + CNF1 culture conditions for Alizarin Red staining and then fixed for 15 min at room temperature with 4% paraformaldehyde, rinsed with double-distilled water (ddH_2_O) and stained for 30 min at room temperature with 0.5% Alizarin Red S solution (pH 4.2). Cells were then thoroughly washed three times with distilled water to reveal the red calcium-containing mineralized nodules. Nodules were imaged using a microscope (Tokyo, Japan), as previously described [31] and quantified using ImageJ software.

### 4.8. Confocal Microscopy

MC3T3-E1 cells (3 × 10^6^) cultured under 1 g, SMG or SMG + CNF1 culture conditions were collected, washed with PBS and incubated for 15 min at 37 °C in the dark with 100 nM MitoTracker Green FM (Life Technologies Inc., Carlsbad, CA, USA) and 5 µg/mL Hoechst 33342 solution (Life Technologies Inc., Carlsbad, CA, USA). Cells were subsequently deposited on microscope slides and imaged using the Zeiss LSM700 confocal microscope (Carl Zeiss, Oberkochen, Germany), as previously described [17,18,19].

### 4.9. Seahorse Assays

A Seahorse XFp analyzer (Seahorse Bioscience, Agilent Technologies, Santa Clara, CA, USA) was used to measure the oxygen consumption rate (OCR) and extracellular acidification rate (ECAR) of MC3T3-E1 cells, according to the manufacturer’s instructions. Cells were plated onto XF8 cell culture microplates (1.5 × 10^5^ cells per well) supplemented with 10 mM glucose, 1 mM sodium pyruvate and 2 mM L-glutamine (Agilent, Lexington, MA, USA). A mitochondrial stress test was performed by measuring basal OCR (pmol/min) and OCR upon injecting 1.5 µM oligomycin (port A), 2.5 µM FCCP (port B) and 0.5 µM rotenone and antimycin-A (port C) (Agilent, Lexington, MA, USA) into their respective ports, as previously described [17,18,19]. Data were analyzed by Seahorse Wave Desktop Software v.2.6.3 (Agilent, Santa Clara, CA, USA).

### 4.10. Statistical Analysis

GraphPad Prism 10.0 software (GraphPad, La Jolla, CA, USA) was used to perform an unpaired, two-tailed *t* test or an analysis of variance for comparison of means as previously described [31]. Values of *p* < 0.05 and <0.01 were considered significant and very significant, respectively.

## Data Availability

Data are contained within the article.

## Abbreviations

ALP	alkaline phosphatase
AMG	aerospace microgravity
AMPK	monophosphate-activated protein kinase
AQP9	aquaporin-9
BMP2	bone morphogenic protein-2
BSA	bovine serum albumin
CNF1	cytotoxic necrotizing factor-1
COL1	type-1 collagen
CPT1A	carnitine palmitoyl transferase-1α
ECAR	extracellular acidification rate
ERK1/2	extracellular signal-regulated kinase-1/2
FAK	focal adhesion kinase
FA	focal adhesion
FACs	focal adhesion complex
FAO	fatty acid oxidation
FBS	fetal bovine serum
FCCP	carbonyl cyanide-4 (trifluoromethoxy) phenylhydrazone
FITC	fluorescein isothiocyanate
GSK3β	glycogen synthase kinase-3β
HU	hindlimb unloading
IAP	intracellular adaptor protein
LEF	lymphoid enhancer-binding factor
MSCs	mesenchymal stem cells
mTORC1	mammalian target of rapamycin complex-1
OBD	osteoblast differentiation
OCR	oxygen consumption rate
OXPHOS	oxidative phosphorylation
PBS	phosphate-buffered saline
pFAK	phosphor FAK
PGC-1α	PPARγ coactivator-1α
PVDF	polyninylidene fluoride
RPM	random positonal machine
SIRT1	silent information regulator of transcription-1
SMG	simulated microgravity
TCF1	T-cell factor-1
